# Methodological Quality of Randomized Clinical Trials of Respiratory Physiotherapy in Coronary Artery Bypass Grafting Patients in the Intensive Care Unit: a Systematic Review

**DOI:** 10.21470/1678-9741-2017-0014

**Published:** 2017

**Authors:** Jaqueline Lorscheitter, Cinara Stein, Rodrigo Della Méa Plentz

**Affiliations:** 1 Graduate Program in Health Sciences, Universidade Federal de Ciências da Saúde de Porto Alegre (UFCSPA), Porto Alegre, RS, Brazil.; 2 Laboratory of Clinical Investigation, Instituto de Cardiologia do Rio Grande do Sul (IC), Porto Alegre, RS, Brazil.; 3 Fundação Universidade de Cardiologia (FUC), Porto Alegre, RS, Brazil.

**Keywords:** Physical Therapy Modalities, Thoracic Surgery, Cardiac Surgical Procedures, Methodology, Systematic Review, Review Literature as Topic, Intensive Care Unit

## Abstract

**Objective:**

To assess methodological quality of the randomized controlled trials of physiotherapy in patients undergoing coronary artery bypass grafting in the intensive care unit.

**Methods:**

The studies published until May 2015, in MEDLINE, Cochrane and PEDro were included. The primary outcome extracted was proper filling of the Cochrane Collaboration's tool's items and the secondary was suitability to the requirements of the CONSORT Statement and its extension.

**Results:**

From 807 studies identified, 39 were included. Most at CONSORT items showed a better adequacy after the statement's publication. Studies with positive outcomes presented better methodological quality.

**Conclusion:**

The methodological quality of the studies has been improving over the years. However, many aspects can still be better designed.

**Table:** 

Abbreviations, acronyms & symbols	
CABG	= Coronary artery bypass grafting
FEV1	= Forced expiratory volume in one second
ICU	= Intensive care unit
PaO_2_	= Partial pressure of oxygen
PEDro	= Physiotherapy evidence database
RCT	= Randomized clinical trials

## INTRODUCTION

The large amount of publications in health care makes professionals have difficulty to stay up to date. Moreover, a great part of the available information does not come from studies with adequate methodological quality, what makes them of little clinical relevance. Incomplete or inadequate publication of information on the study planning and driving affects the identification of possible methodological errors, also hampering the use of its findings by the interested parties, since they cannot critically assess its clinical applicability^[1,2]^.

Even though randomized clinical trials (RCT) are gold standard for the assessment of health interventions, this type of study is also prone to bias whether due to researchers arbitrariness when selecting the sample and gauging the analyzed variables, or due to the difficulty of controlling other factors that may influence the clinical outcome. Bias or systematic error can be defined as any tendentiousness in the collection, analysis, interpretation, publication or revision of data, which induces conclusions that systematically tend to distance themselves from the truth^[3]^.

In phase I of cardiac rehabilitation, physiotherapy has an increasingly important role in contributing to the patients return to their social and professional activities in the best possible clinical conditions, thus improving the quality of life^[4]^. In the early postoperative period after a coronary artery bypass grafting (CABG), respiratory physiotherapy has been widely requested in order to reverse or minimize postoperative pulmonary complications^[5]^. Techniques that can improve respiratory mechanics, lung re-expansion and bronchial hygiene are applied, contributing to the patients proper ventilation^[6]^.

Numerous studies over the past decade have documented that physiotherapists are in favor of evidence-based medicine and recognize the importance of using research results to achieve a more scientific-based clinical practice. Therefore, the number of publications that consistently support the best physiotherapy procedures to be followed have been increasing^[7]^. Assessments of physiotherapy intervention studies demonstrate an upward curve in relation to the enrichment of the methodological quality over the past decades^[8-11]^. However, there is still great potential for improvement in their elaboration and development.

It should be noted that no evidence can be observed on the methodological quality of RCT of physiotherapy intervention on CABG postoperative patients in the intensive care unit (ICU). Therefore, this research is needed since the fulfillment or not of the criteria for a correct development of this research design can influence the results. Also, complementarily, the dissemination of these data will stimulate further research to be developed with a superior methodological quality, showing the main points that should be better outlined and planned. It will then be possible to obtain greater benefits, as well as improved outcomes for critical patients in daily clinical practice. It should be noted that there is no evidence on the methodological quality of RCT of physiotherapy intervention on CABG postoperative patients in the Intensive Care Unit (ICU).

## METHODS

This review was conducted in accordance with the recommendations proposed by the Cochrane Collaboration and the Preferred Reporting Items for Systematic Review and Meta-analyses: The PRISMA^[12,13]^. The studies methodological quality was evaluated using the Cochrane Collaboration's tool for assessing risk of bias^[12]^, and the correct description of the RCT's items was evaluated using the CONSORT Statement^[14]^ and its extension for clinical trials of nonpharmacologic treatment interventions^[15]^. When certain items were not applicable to all studies (as in the case of the evaluation of multicenter studies), they were considered as adequate.

### Eligibility Criteria

Studies designed as RCT's, with respiratory physiotherapy intervention, associated or not with neuromusculoskeletal physiotherapy, in postoperative patients of CABG in the ICU were included. Studies whose intervention also happened in the preoperative period were included as well. The following were ineligible for inclusion in the review: studies whose patients had undergone another associated surgery and studies that did not contain terms related to physiotherapy and its synonyms (physiotherapy, physical therapy, physiotherapists, physical therapists, and respiratory therapists) anywhere in the paper.

### Search Strategies

The search was conducted in the following electronic databases (from inception to May 26, 2015): MEDLINE (via PubMed), Central Register of Controlled Trials (Cochrane CENTRAL) and Physiotherapy Evidence Database (PEDro). Additionally, manual search was conducted in the references of published papers. The search terms used were "Coronary Artery Bypass Grafting", terms related to respiratory physiotherapy interventions, such as "breathing exercises" and "respiratory muscle training", and a word sequence with high sensitivity for the search of randomized RCT described by Robinson & Dickersin^[16]^. Papers not published in English were excluded. The full search strategy used in the PubMed, which was adjusted for the search in the other databases, is shown in Table 1.

**Table 1:** Strategy used for PubMed.

#1	"Coronary Artery Bypass"[Mesh] OR "Coronary Artery Bypass Grafting" OR "Coronary Artery Bypass Surgery" OR "Bypass, Coronary Artery" OR "Artery Bypass, Coronary" OR "Artery Bypasses, Coronary" OR "Bypasses, Coronary Artery" OR "Coronary Artery Bypasses" OR "Aortocoronary Bypass" OR "Aortocoronary Bypasses" OR "Bypass, Aortocoronary" OR "Bypasses, Aortocoronary" OR "Bypass Surgery, Coronary Artery" "Myocardial Revascularization"[Mesh] OR "Myocardial Revascularizations" OR "Revascularization, Myocardial" OR "Revascularizations, Myocardial" OR "Internal Mammary Artery Implantation"
#2	"breathing exercises" OR "Intermittent Positive Pressure Breathing" OR "continuous positive airway pressure" OR "weaning from mechanical ventilation" OR "mechanical ventilation" OR "noninvasive ventilation" OR "breathing exercises" OR "Exercise, Breathing" OR "Respiratory Muscle Training" OR "Muscle Training, Respiratory" OR "Training, Respiratory Muscle" OR "Breathing, Intermittent Positive Pressure" OR "Intermittent Positive Pressure Breathing" OR "Positive Pressure Breathing, Intermittent" OR "Intermittent Positive Pressure Breathing (IPPB) " OR "Inspiratory Positive Pressure Breathing" OR "Breathing, Inspiratory Positive Pressure" OR "Inspiratory Positive Pressure Breathing" OR "Positive Pressure Breathing" OR "Inspiratory" OR "IPPB" OR "CPAP Ventilation" OR "Ventilation, CPAP" OR "Biphasic Continuous Positive Airway Pressure" OR "Bilevel Continuous Positive Airway Pressure" OR "Nasal Continuous Positive Airway Pressure" OR "nCPAP Ventilation" OR "Ventilation, nCPAP" OR "Airway Pressure Release Ventilation" OR "APRV Ventilation Mode" OR "APRV Ventilation Modes" OR "Ventilation Mode, APRV" OR "Ventilation Modes, APRV" OR "Respiration, Artificial" OR "Artificial Respiration" OR "Artificial Respirations" OR "Respirations, Artificial" OR "Ventilation, Mechanical" OR "Mechanical Ventilations" OR "Ventilations, Mechanical" OR "Mechanical Ventilation" OR "Noninvasive Ventilations" OR "Ventilation, Noninvasive" OR "Ventilations, Noninvasive" OR "Non-Invasive Ventilation" OR "Non-Invasive Ventilations" OR "Ventilation, Non-Invasive" OR "Ventilations, Non-Invasive" OR "Non Invasive Ventilation" OR "Non Invasive Ventilations" OR "Ventilation, Non Invasive" OR "Ventilations, Non Invasive"
#3	randomized controlled trial[pt] OR controlled clinical trial[pt] OR randomized controlled trials[mh] OR random allocation[mh] OR double-blind method[mh] OR single-blind method[mh] OR clinical trial[pt] OR clinical trials[mh] OR ("clinical trial"[tw]) OR ((singl*[tw] OR doubl*[tw] OR trebl*[tw] OR tripl*[tw]) AND (mask*[tw] OR blind*[tw])) OR ("latinsquare"[tw]) OR placebos[mh] OR placebo*[tw] ORrandom*[tw] OR research design[mh:noexp] OR follow-up studies[mh] OR prospective studies[mh] OR cross-over studies[mh] OR control*[tw] OR prospectiv*[tw] OR volunteer*[tw]) NOT (animal[mh] NOT human[mh]).

### Study Selection and Data Extraction

The selection of studies was carried out by two reviewers (J.L. and C.S.), independently, in two stages:

I - selection of studies by reading the titles and abstracts;
II - full analysis of papers selected in Phase I.

Papers were included in accordance with the eligibility criteria specified previously. In case of disagreement on the paper's inclusion and with no consensus between the reviewers, a third reviewer (R.P.) was consulted. The primary outcome extracted was proper fulfilling of the Cochrane Collaboration's tool's items, and the secondary outcome extracted was suitability to the requirements of the CONSORT Statement and its extension. The data extraction was performed separately and independently by both reviewers (J.L. and C.S.) and cross-checked. Disagreements regarding the data extraction were solved by a third author (R.P.). Three standardized forms were used, which contained: the 25 items of the CONSORT checklist, the 7 items of the Cochrane Collaboration's tool for assessing risk of bias, and the 16 items of the CONSORT checklist extension for clinical trials of non-pharmacologic treatment interventions. For the CONSORT Statement items, the concept of "adequate" or "inadequate" was assigned, according to the description or not of each item in the checklist. The Cochrane Collaboration's tool's items without a clear description were classified with the word "no" or "not report". In the case of missing data, the authors were contacted by e-mail at least twice. The study was excluded if the data were still insufficient after this process.

### Data Analysis

The results are going to be descriptively displayed (frequency and percentage).

## RESULTS

### Description of Studies

**Fig. 1:**
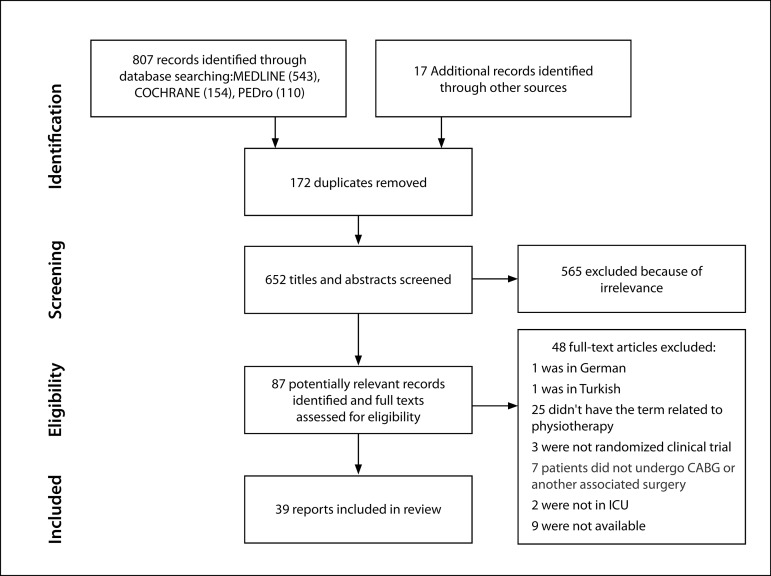
Study flowchart.

The search strategy identified 807 potentially relevant studies, adding a further 17 studies drawn from the reference lists. Subsequently, 172 duplicates were discarded and 565 irrelevant studies were excluded. Among the 87 resulting records, two were excluded for not having been published in English, 25 had not described a term related to physiotherapy and its synonyms, three were not RCT's, seven were not with postoperative patients of CABG or had other associated surgery, two studies had not been performed in the ICU and nine studies were not available. Figure 1 shows the study flowchart.

CABG=coronary artery bypass grafting; ICU=intensive care unit

Among the 39 studies included^[17-55]^, 41.02% (n=16) were conducted in Brazil, 56.41% (n=22) were published between 2000 and 2010, and only 12.82% (n=5) were published in journals specialized in physiotherapy. In relation to the sample, in 33.33% (n=13) of the studies the number of patients was higher than 70, in 58.97% (n=23) the average age was of over 60 years, and in 84.61% (n=33) of the studies more than half of the sample consisted of males. The treatment was provided only in the postoperative period in 69.23% (n=27) of the studies, and in 51.28% (n=20) a patients were monitored until discharge.

The most widely used techniques were re-expansive ventilatory exercises (56.41%), ventilatory exercises for bronchial hygiene (48.71%) and non-invasive mechanical ventilation (41.02%). There was an association of techniques in 69.23% (n=27) of the studies.

The most researched outcomes were atelectasis (48.71%), forced expiratory volume in one second (FEV1) (41.02%), invasive mechanical ventilation time (35.89%) and partial pressure of oxygen (PaO_2_) (35.89%). The Table 2 shows the characterization studies.

**Table 2:** Characteristics of studies included in systematic review.

Author, Year	Intervention	Comparator	N(IG/CG)	Mean Age± SD (IG/CG)	Male (IG/CG)	Protocol	Conclusion
Al Jaaly et al.^[17]^, 2013	BPAP, RVE, VEBH, EM, IS, NBL	RVE, VEBH, EM, IS, NBL	66 / 63	65.7±10.7 / 69.4±8.86	Not reported	IG: Usual care and BPAP during the first 24 hours after extubation CG: usual care alone twice per day for the first 2 to 3 days after surgery Outcomes: atelectasis, FEV1, ICU days, days of hospitalization, PaCO_2_	Among patients undergoing elective coronary artery bypass grafting, the use of BPAP at extubation reduced the recovery time. Supported by trained staff, more than 75% of all patients allocated to BPAP tolerated it for more than 10 hours
Barros et al.^[18]^, 2010	IMT, TM, PD, AT	TM, PD, TS	23 / 15	62.1±8.1 / 67±7.1	19 / 6	IG: conventional physiotherapy and IMT, with three sets of ten repetitions, once a day, with 40% of the PI_max_ CG: Conventional physiotherapy with four sets of six cycles of vibrocompression associated with postural drainage and aspiration where necessary, twice a day Outcomes: PI_max_, PE_max_, Dyspnea, pain, PEF, CV	There is loss of respiratory muscle strength in patients undergoing coronary revascularization. The IMT, performed in the postoperative period, was effective in restoring the following parameters: PI_max_, PE_max_, PEF and CV in this population
Blattner et al.^[19]^, 2008	MH, TS	TS	28 / 27	55.6±8.7 / 57.6±4.9	16 / 17	IG: Twenty minutes MH with flow of 15 l/ m and expiratory pressure 10 cmH_2_O, often 18 to 30 rpm and TS CG: TS Outcomes: atelectasis, pleural effusion, consolidation, PaO_2_, Cstat, IMV time, days of hospitalization	The group that received early MH had markedly better oxygenation and static compliance as well as shorter mechanical ventilation times than the control group. The length of hospital stay and incidence of postoperative pulmonary complications were similar in the two groups
Borges et al^[20]^, 2013	WP with PEEP (5 cmH_2_O)	WP with PEEP (8 cmH_2_O) / WP with PEEP (10 cmH_2_O)	44 / 47 / 45	20<60>24 / 22<60>25 / 19<60>26	29 / 32 / 35	IG: PEEP 5 cmH_2_O after ICU admission and extubation when met clinical conditions CG: PEEP 8 cmH_2_O after ICU admission and extubation when met clinical conditions CG: PEEP 10 cmH_2_O after ICU admission and extubation when met clinical conditions Outcomes: ventilatory mechanics, pulmonary shunt, oxygenation index	Higher levels of positive end-expiratory pressure in immediate postoperative period of coronary artery bypass grafting improved pulmonary compliance values and increased oxygenation indexes, resulting in lower frequency of hypoxemia
Borghi-Silva et al.^[21]^, 2005	PEP, TM, TS,VEBH, RVE, EULL, AM, LA	TM, TS, VEBH, RVE, EE, EULL, AM, LA	8 / 16	59.9±9.8 / 55.9±11.9	Not reported	IG: two daily sessions of about 40 minutes. TM, TS, VEBH, EE, AM, LA. PEP through facial mask with PEEP 10 cmH_2_O CG: two daily sessions of about 40 minutes. TM, TS, VEBH, EE, AM, LA Outcomes: VC, FVC, length of stay, PI_max_, PE_max_.	These data suggest that cardiac surgery produces a reduction in inspiratory muscle strength, pulmonary volume, and flow. The association of positive expiratory pressure with physiotherapy intervention was more efficient in minimizing these changes, in comparison to the physiotherapy intervention alone. However, in both groups, the pulmonary volumes were not completely reestablished by the fifth postoperative day, and it was necessary to continue the treatment after hospital convalescence
Castellana et al.^[22]^, 2003	WP with VC-CMV	WP with PC-CMV	32 / 29	65±7 / 64±11	Not reported	IG: IMV in the VC-CMV mode with volume of 7 ml/kg respiratory rate of 12 and PEEP 5 cmH_2_O, inspiratory time of 33% and 60% FiO_2_ CG: IMV in the PC-CMV mode with volume of 7 ml / kg respiratory rate of 12 and PEEP 5 cmH_2_O, inspiratory time of 33% and 60% FiO_2_ Outcomes: shunt, oxygenation index	Ventilatory modes controlled the volume and pressure were equally effective in treating hypoxemia observed in patients in the postoperative immediate coronary artery bypass surgery, showing that the pattern of administration of inspiratory flow. It is of little relevance for the treatment of postoperative hypoxemia
Celebi et al.^[23]^, 2008	AR, NIV, VEBH, EM, IS	NIV, VEBH, EM, IS / AR, VEBH, EM, IS / VEBH, EM, IS	25 / 25 / 25 / 25	52±9 / 57±8 / 58±6 / 57±7	20 / 18 / 21 / 22	IG: NIV through facial mask for periods of one hour, starting 6h after extubation in the first 24 hours, in the SP mode around 10 cmH_2_O, PEEP 5 cmH_2_O and FiO_2_ 40%. VEBH, EM and IS after extubation IG: AR in CPAP mode with peak pressure 40 cmH_2_O (20 cm H_2_O inspiratory pressure and 20 cmH_2_O PEEP) sustained for 30 seconds and FiO_2_ by 40%. VEBH, EM and IS after extubation. IG: application of NIV and AR as the two previously described protocols. VEBH, EM and IS after extubation CG: maintenance of 5 cmH_2_O PEEP during the IMV and VEBH, EM and IS after extubationOutcomes: pleural effusion, atelectasis, VC, FEV1	NIV associated with AR provided better oxygenation both during and after the mechanical ventilation period. NIV either alone or in combination with AR provided lower atelectasis scores and better early pulmonary function tests compared to the control group, without a significant difference regarding the duration of mechanical ventilation, intensive care unit stay, and the length of hospitalization. NIV combined with AR is recommended after open heart surgery to prevent postoperative atelectasis and hypoxemia
Crowe and Bradley^[24]^, 1997	IS, RVE, VEBH, TM, EM, TS, EULL, AM, PE	RVE, VEBH, TM, EM, TS, EULL, AM, PE	90 / 95	64±8.9 / 64.8±8.6	74 / 79	IG: PE, RVE, TM, VEBH and TS once or twice a day. After extubation, EULL and AM. Spirometry incentive driven volume used every hour by the patient CG: PE, RVE, TM, VEBH and TS once or twice a day. After extubation, EULL and AM Outcomes: atelectasis, pulmonary congestion, pneumothorax, pleural effusion, FEV1, FVC, respiratory infection, SpO_2_, days of hospitalization	Incentive spirometry combined with physical therapy is no more effective than postoperative physical therapy alone in reducing atelectasis for this population. Use of the spirometer, however, was not monitored, and although the study mimicked practice as it often occurs, the effectiveness of the spirometer cannot be fully evaluated
Dongelmans et al.^[25]^, 2009	WP with SA	WP with PC-CMV and SP	64 / 64	65±9 / 67±8	56 / 51	IG: ventilation adapted support, minute volume of 100% of the predicted weight, FiO_2_ 50%, PEEP 10 cmH_2_O, trigger the 2 L/s CG: PC-CMV, CV 6-8 ml/kg, respiratory rate of 12-15 rpm, FiO_2_ 50%, PEEP 10 cmH_2_O, 2 L/s trigger. After spontaneous ventilation with SP 10 cmH_2_O, trigger 2 L/s, expiratory sensitivity 25% and rise time 50 ms. Outcomes: days of ICU, length of stay, PaO_2_, PaCO_2_, IMV Time	Weaning automation with SA is feasible and safe in non-fast-track coronary artery bypass grafting patients. Time until tracheal extubation with SA equals time until tracheal extubation with standard weaning and allows for frequent (automatic) switches between controlled and assisted ventilation
El-Kader^[26]^, 2011	RVE, VEBH, TM, IS	RVE, VEBH, TM, CPAP / RVE, VEBH, TM, RPPI	12 / 12 / 12	48.7±6.8 / 47.4±6 / 49.6±7.1	Not reported	IG: 3-5 RVE followed 2-3 VEBH at least 10 times in 15 minutes. If necessary, positioning and thoracic maneuvers. IS volume for five minutes, five times a day IG: 3-5 RVE followed 2-3 VEBH at least 10 times in 15 minutes. If necessary, positioning and thoracic maneuvers. CPAP 10 cmH_2_O for 15 minutes daily CG: 3-5 RVE followed 2-3 VEBH at least 10 times in 15 minutes. If necessary, positioning and thoracic maneuvers. RPPI with inspiratory phase of 20%, peak inspiratory pressure of 15 cmH_2_O for 15 minutesOutcomes: PaCO_2_, PaO_2_	Incentive spirometry in addition to the usual respiratory physical therapy is recommended for patients in phase I of cardiac rehabilitation program after CABG
Ferreira et al.^[27]^, 2010	IS, PEP	RVE, VEBH, EM	8 / 8	61±2 / 60±3	6 / 6	IG: IS volume coupled to a PEP valve after extubation, with increased expiratory pressure progressively to 5 cmH_2_O to 15 cmH_2_O twice a day with supervision and twice a day without supervision, lasting 15 minutes CG: guidance on VEBH, EM and RVE Outcomes: FVC, FEV1, PEF, PI_max_, PE_max_, 6MWD, Evaluation level of physical activity, evaluation of quality of life	Patients undergoing IS + PEP presented less dyspnea and lower sensation of effort after SMWT and also better quality of life 18 months after CABG
Franco et al.^[28]^, 2011	BPAP, RVE, VEBH, TM, EULL, PE	RVE, VEBH, TM, EULL, PE	13 / 13	Not reported	10 / 7	IG: RVE, VEBH, TM, EULL, PE for two days after surgery. BPAP in the spontaneous mode with inspiratory pressure of 8-12 cmH_2_O and expiratory of 6 cmH_2_O, twice daily for 30 minutes CG: RVE, VEBH, TM, EULL, PE for two days after surgery Outcomes: CV, MV, VC, PI_max_, PE_max_, PEF	Coronary artery bypass surgery leads to deterioration of respiratory function postoperatively, and the application the BPAP may be beneficial to restore lung function more quickly, especially vital capacity, safely, and well accepted by patients due to greater comfort with the feeling of pain during the execution of respiratory therapy
Garcia and Costa^[29]^, 2002	IMT (twice a day)	IMT (once a day) / VEBH	20 / 20 / 20	56±11 / 58±7.5 / 63±9	13 / 16 / 11	IG: three sets of 10 repetitions, twice a day. Efforts inspiratory in a free load manometer for at least five seconds IG: three sets of 10 repetitions daily. Efforts inspiratory in a free load manometer for at least five seconds CG: conventional treatment, especially VEBH Outcomes: PI_max_, PE_max_, PEF, cytometry	It was found that through a specific IMT was increased respiratory muscle strength both the group that trained two as in trained once a day, compared to the control group which had no change
Gust et al.^[30]^, 1996	RCP	CPAP / BPAP	25 / 25 / 25	60.5±7.5 / 63±7 / 62.6±7.5	23 / 21 / 23	IG: oxygen therapy by NC 6l m and RCP IG: CPAP with 7.5 cmH_2_O and FiO_2_ of 50% CG: BPAP with 10 cmH_2_O and PEEP 5 cmH_2_O, getting oxygen to 10 l/ m Outcomes: cardiac index, pulmonary blood volume index, extravascular water content	Mask CPAP and nasal BPAP after extubation of the trachea prevent the increase in extravascular lung water during weaning from mechanical ventilation. This effect is seen for at least 1 h after the discontinuation of CPAP or BPAP treatment
Haefener et al.^[31]^, 2008	IS, PEP	RVE, VEBH, EM	17 / 17	62±6 / 60±7	14 / 14	IG: IS volume associated with PEP twice a day 15-20 minutes with expiratory pressure increased progressively 2.5 cmH_2_O the 15 cmH_2_O CG: patients were educated about VEBH, EM and RVE Outcomes: plethysmography, 6MWD, atelectasis, pleural effusion, consolidation, FVC, FEV1, IMV time	In patients undergoing CABG, IS + PEP results in improved pulmonary function and 6-minute walk distance as well as a reduction in postoperative pulmonary complications
Hendrix et al.^[32]^, 2006	WP with PRVC, VS	WP with PRVC, SIMV, CPAP	10 / 10	54±9 / 66±4	10 / 10	IG: WP with PRVC and activated automatic mode function triggered when the patient's ventilatory cycle the mode automatically changed to VSV CG: WP with PRVC when the patient triggered a ventilation cycle, the team modified to SIMV mode with frequency of 5 and PS 10 cmH_2_O. When patients become fully alert, they were changed to the mode CPAP 10 cmH_2_O Outcomes: VC, FEV1, PaO_2_, PaCO_2_, IMV time	Automode ventilator weaning trended toward more rapid extubation than did conventional protocol driven ventilation in conjunction with a standardized weaning protocol. Physiologic and hemodynamic factors were better in patients using automode ventilation compared to patients using conventional ventilation. Automode ventilation was well tolerated and did not induce significant adverse effects.
Herdy et al.^[33]^, 2008	RPPI, RVE, IS, AM, LA	NPI	29 / 27	61±10 58±9	20 / 20	IG: RPPI, RVE, IS, AM, LA five days before surgery and continuing after extubation to discharge. Energy expenditure was 2 METS, progressing up to 4 METS CG: NIP Outcomes: pleural effusion, atelectasis, ICU days, hospital days, PEF, IMV time, 6MWD	Pre- and postoperative cardiopulmonary rehabilitation in patients who await CABG in the hospital is superior to standard care and leads to a reduced rate of postoperative complications and shorter hospital stay
Hirschhorn et al.^[34]^, 2008	VEBH, PD, AM, LA, PE	PWC / IS, EULL, AM	31 / 31 / 30	63.6±8.5 / 63.2±10.8 / 61.8±7.2	26 / 27 / 27	IG: five sets of 4 repetitions of IS during the service and guidance to perform every hour. RVE and EMSI IG: PWC CG: PE, PD, RVE, VEBH, LA. AM starting at 10 m up to 30 m in the morning and night Outcomes: 6MWD, VC, quality of life, atelectasis, injury, failure or pulmonary consolidation	A physiotherapy-supervised, moderate intensity walking program in the inpatient phase following CABG improves walking capacity at discharge from hospital. The performance of respiratory and musculoskeletal exercises confers no additional benefit to the measured outcomes
Jenkins et al.^[35]^, 1989	RVE, VEBH, EULL, AM, LA, PE, TM	VEBH, EULL, AM, LA, PE / VEBH, EULL, AM, LA, PE, IS	35 / 38 / 37	55±8.5 / 56±6.9 /54±7.6	35 / 38 / 37	IG: Guidance on VEBH, EULL, AM, LA, PE. Three to five repetitions of RVE and if necessary were carried TM. At least 10 RVE every hour until the fifth day after surgery IG: Guidance on VEBH, EULL, AM, LA, PE. Three to five repetitions of IS. At least 10 reps every hour until the fifth day after surgery CG: Guidance on VEBH, EULL, AM, LA, PE Outcomes: FEV1, PEF, FVC, consolidation, PaCO_2_, PaO_2_, pain	It is concluded that the addition of breathing exercises or incentive spirometry to a regimen of early mobilization and huffing and coughing confers no extra benefit after uncomplicated coronary artery bypass grafting
Johnson et al.^[36]^, 1995	RVE, EM	RVE, SMI, EM (group with minimal atelectasis) / RVE, SMI, EM (group marked atelectasis) / RVE, SMI, TM, EM	48 / 49 / 64 / 63	60±10 / 64±11 / 66±8 / 64±11	39 / 40 / 52 / 53	IG: EM and five repetitions of RVE every hour IG: group with minimal atelectasis. EM and five repetitions of SMI starting from the residual functional capacity to total lung capacity IG: group marked atelectasis. EM and five repetitions of SMI starting from the residual functional capacity to total lung capacity CG: During the operation of SMI, application TM with frequency of 1-2 per second and EM. Three daily sessions. Outcomes: atelectasis, VC, FVC, FEV1, days of hospitalization, PI_max_, PE_max_, pain	We concluded that postoperative respiratory dysfunction is common but does not commonly cause significant morbidity or prolong hospital stay. Adding SMI to patients with minimal atelectasis at extubation does not improve clinical outcomes. Similarly, adding TM to patients with marked atelectasis does not improve outcomes over those obtained with SMI and early ambulation
Marvel et al.^[37]^, 1986	WPwith ambient pressure	WP with PEEP of 5 cmH_2_O, CPAP of 5 cmH_2_O / WP with PEEP 10 cmH_2_O	17 / 15 / 12	62.7±1.7 / 60.9±2.9 / 55.8±2.7	Not reported	IG: WP pressure environment IG: WP with 5 cmH_2_O and CPAP 5 cmH_2_O for an hour and a half before extubation CG: WP with 10 cmH_2_O and CPAP 5 cmH_2_O for half an hour before extubation Outcomes: atelectasis, days of hospitalization, PaO_2_	We conclude that routine PEEP improves pulmonary oxygen transfer but, once discontinued, PEEP offers no sustained beneficial effect upon impaired oxygen transfer or roentgenographic evidence of atelectasis following CABG
Matheus et al.^[38]^, 2012	IMT, RVE, IS, AM, PE	RVE, IS, AM, PE	23 / 24	61.8±13.5 / 63.3±10.2	18 / 16	IG: RVE, IS, AM, PE and IMT twice a day with three sets of 10 repetitions with 40% of MIP CG: RVE, IS, AM, PE Outcomes: PI_max_, PE_max_, CV, VC, PEF, pleural effusion, atelectasis, ICU days, hospital days, IMV time	Patients undergoing cardiac surgery suffer reduction of VC and respiratory muscle strength after the surgery. The muscle training performed was effective in recovering the CV and VC in PO3, the trained group
Matte et al.^[39]^, 2000	VEBH, EM, IS, NBL	RVE, EM, IS, NBL, CPAP / VEBH, EM, IS, NBL, BPAP	30 / 33 / 33	63±8 / 65±8 / 64±9	25 / 30 / 30	IG: Routine physiotherapy (VEBH, NBL, EM and IS)IG: Routine physiotherapy, CPAP 5 cmH_2_O CG: Routine physiotherapy, BPAP with the inspiratory pressure 12 cmH_2_O and expiratory pressure 5 cmH_2_O Outcomes: VC, FEV1, PaO_2_, PaCO_2_, atelectasis, days of ICU	We conclude that preventive use of NIV can be considered as an effective means to decrease the negative effect of coronary surgery on pulmonary function
Mendes et al.^[40]^, 2010	RVE, VEBH, PD, TM, EM, PE, EE, EULL, AM, LA	RVE, VEBH, PD, TM, EM, PE	24 / 23	60±8 / 58±9	16 / 20	IG: Four sets of 10 repetitions of RVE and VEBH once daily. If necessary, PD and TM CG: Four sets of 10 repetitions of RVE and VEBH once daily. If necessary, PD and TM. EE with five sets of 10 repetitions, EULL with two sets of 15 reps, 10 minutes AM, LA-four steps Outcomes: ICU days, hospital days, IMV time, heart rate and RR interval	Short-term supervised physiotherapy exercise protocol during inpatient cardiac rehabilitation improves cardiac autonomous regulation at the time of discharge. Thus, exercise-based inpatient cardiac rehabilitation might be an effective non-pharmacological tool to improve autonomic cardiac tone in patient's post-CABG
Michalopoulos et al.^[41]^, 1998	WP with ZEEP	WP with PEEP of 5 cmH_2_O / WP with PEEP of 10 cmH_2_O	22 / 24 / 21	61.1±6.1 / 60.9±6.2 / 61.9±6.6	18 / 20 / 16	IG: ZEEP during IMV postoperatively until extubation IG: PEEP 5 cmH_2_O during IMV postoperatively until extubation CG: PEEP 10 cmH_2_O during IMV postoperatively until extubation Outcomes: atelectasis, IMV time, oxygenation index, cardiac index	We concluded that low levels of PEEP have no advantage over zero PEEP in improving gas exchange in the early postoperative course of patients following open heart surgery
Muller et al.^[42]^, 2006	CPAP	RPPI	20 / 20	61±5.8 / 62.1±7.3	16 / 17	IG: CPAP to 5 cmH_2_O and 3 l/m oxygen within 3 hours for 15 minutes every hour, on the 24^th^ and 48^th^postoperative for 30 minutes in two 15-minute sets CG: RPPI 20 cmH_2_O the 30 cmH_2_O with serum as diluent in the micronebulizer. Within 3 hours for 15 minutes every hour, on the 24^th^ and 48^th^ postoperative for 30 minutes in two 15-minute sets Outcomes: PaO_2_, PaCO_2_, dyspnea, ventilometry	Both devices were shown to be able to keep pO_2_, pCO_2_, and SPO_2_ values within normal limits. However, when the objective was pulmonary reexpansion with less imposed workload, the Müller resuscitator was more effective because of its prompter action and consequently lower levels of dyspnea, respiratory rate (RR) and use of accessory muscle were observed
Oikkonen et al.^[43]^, 1991	IS, PE	RPPI, PE	26 / 26	55±1 / 55±1	22 / 22	IG: PE with guidance on RVE, VEBH. IS volume with 3 seconds support at least 5 times per training CG: PE with guidance on RVE, VEBH. RPPI with a peak pressure of 10 to 15 cmH_2_O pressure for not less than 4 daily sessions Outcomes: atelectasis, congestion, pleural effusion, diaphragm elevation, VC, PEF, PaO_2_, PaCO_2_	Based on the three variables studied, we consider both devices equal in efficiency after coronary surgery
Renault et al.^[44]^, 2009	RVE, VEBH, EM, NIV	VEBH, EM, IS, NIV	18 / 18	54.8±7.4 / 58.7±9.2	13 / 16	IG: EM, VEBH, NIV with two pressure levels for 30 minutes twice a day in the ICU and once in the inpatient unit. RVE three sets of ten repetitions CG: EM, VEBH, NIV with two pressure levels for 30 minutes twice a day in the ICU and once in the inpatient unit. IS three sets of ten repetitions with the position of the adjustment ring 0-2, prioritizing slow flows Outcomes: PI_max_, PE_max_, FVC, FEV1, IMV time	No significant differences were observed in maximal respiratory pressures, spirometric variables and oxygen saturation in patients undergoing deep breathing exercises and incentive spirometry in postoperative coronary artery bypass surgery
Richter Larsen et al.^[45]^, 1995	RVE, VEBH, EM, AM, PE, PEP	RVE, VEBH, EM, AM, PE, IR, PEP / RVE, VEBH, EM, AM, PE	Not reported	Not reported	Not reported	IG: Twice a day RVE, VEBH and EM. PEP with 10-15 cmH_2_O IG: Twice a day RVE, VEBH and EM. IR around 20 cmH_2_O and PEP of 10-15 cmH_2_O CG: twice a day RVE, VEBH and EM Outcomes: Atelectasis, FVC, PaO_2_	We did not find any significant difference among the three groups; however, a tendency to decreased risk of having post operative complications was observed in the groups having positive expiratory pressure and inspiratory resistance-positive expiratory pressure
Romanini et al.^[46]^, 2007	RPPI	IS	20 / 20	56.4±8.8 / 57.1±9.8	12 / 16	IG: RPPI for ten minutes, five minutes interval and again applied for ten minutes CG: IS volume for ten minutes, five minutes interval and again applied for ten minutes Outcomes: FEV1, Tiffenau index, PI_max_, PE_max_, ventilometry	In order to reverse hypoxemia earlier, the RPPI was more efficient compared to IS; however, to improve the strength of respiratory muscles, it was more effective
Savci et al.^[47]^, 2011	IMT, RVE, VEBH, EM, EULL, AM, LA	RVE, VEBH, EM, EULL, AM, LA	22 / 21	62.8±8.6 / 57.4±1.4	19 / 19	IG: IMT twice a day for ten days (five before and five postoperatively), EM, EULL, RVE, VEBH, AM, LA CG: EM, EULL, RVE, VEBH, AM, LA Outcomes: atelectasis, pleural effusion, consolidation, FVC, FEV1, Tifennau index, PI_max_, PE_max_, 6MWD, quality of life	IMT results in faster recovery of inspiratory muscle strength, functional capacity, intensive care unit stay, quality of life and psychosocial status after CABG
Savci et al.^[48]^, 2006	RVE, VEBH, EM, EULL, AM, CAR	RVE, VEBH, EM, EULL, AM, IS	30 / 30	55.2±8.5 / 57.2±8.9	30 / 30	IG: RVE, VEBH, EM, EULL. AM 30 and 80m in the morning and afternoon on the first postoperative day. On the second day, AM for five minutes. On the third day, the walk was free in the hallway. CAR consisted of 1-2 controlled breaths, followed 3 RVE inspiratory pause of 3 seconds, controlled breaths 1-2 VEBH CG: RVE, VEBH, EM, EULL. AM 30 and 80 m in the morning and afternoon on the first postoperative day. On the second day, AM for five minutes. On the third day, the walk was free in the hallway. IS was applied followed by 3 repetitions inspiratory pause of 3 seconds. VEBH 1-2 controlled breaths. By the second day after surgery, two daily sessions and after, once a day, 15 minutes session Outcomes: VC, FVC, FEV1, PEF, 6MWD, atelectasis, congestion, infiltration, pneumothorax, pleural effusion, pulmonary edema, pain	Both treatments improved arterial oxygenation from the first day of the postoperative period. After a 5-day treatment, functional capacity was well preserved with the usage of CAR or IS Both physiotherapy methods had similar effects on the rate of atelectasis, pulmonary function, and pain perception
Stein et al.^[49]^, 2009	Medical consultation and nursing , RVE, VEBH, EE, EULL, AM, LA, PE, PEP	PE, medical consultation and nursing	10 / 10	64±7 / 63±6	6 / 5	IG: Medical consultation and nursing, PE, VEBH, RVE, EE, EULL, AM, LA, PEP with progressive pressure 3-8 cmH_2_O for 3-12 minutes CG: PE, medical visits and nursing Outcomes: FVC, FEV1, PI_max_, PE_max_, SMWT, dyspnea, IMV time	A 6-day rehabilitation program attenuated the postoperative reduction in respiratory muscle strength and also improved the recovery of functional capacity after CABG. The correlation between PI_max_ and VO_2_ peak during the late postoperative period suggests that inspiratory muscle strength is an important determinant of functional capacity after CABG
Stiller et al.^[50]^, 1994	PE and RVE, VEBH, TM, PD (twice a day)	PE and RVE, VEBH, TM, PD (four times a day) / NPI	40 / 40 / 40	61±9 / 63±8 / 62±11	33 / 32 / 33	IG: PE, RVE, VEBH (2x / day for the first two days of PO and e 1x / day 3 and 4 PO. 3-5 EVR followed 2-3 HB (also independently every hour). If necessary TM and PD IG: PE, RVE, VEBH (4x / day for the first 2 days of PO and 2x / day 3 and 4 PO. 3-5 RVE followed 2-3 VEBH (also independently every hour) if necessary TM and PD CG: No physical therapy intervention in the pre- or postoperative period Outcomes: FVC, days of hospitalization, IMV time, PaO_2_, PaCO_2_, oxygenation index	The necessity for prophylactic chest physiotherapy after routine coronary artery surgery should be reviewed
Sulzer et al.^[51]^, 2001	WP with SA	WP with SIMV	16 / 20	59.2±8.7 / 59.7±8.1	12 / 14	IG: WP with SA 100% of minute ventilation, 100% FiO_2_, 4 cmH_2_O PEEP, peak pressure 25 cmH_2_O and sensitivity 2l / min CG: WP with SIMV, tidal volume of 7 ml / kg, decelerating flow, respiratory rate of 12, 100% FiO_2_, PEEP 4 cmH_2_O, sensitivity 2l / min Outcomes: days of ICU, IMV time, PaO_2_, oxygenation index	A respiratory weaning protocol based on SA is practicable; it may accelerate tracheal extubation and simplify ventilatory management in fast-track patients after cardiac surgery. The evaluation of potential advantages of the use of such technology on patient outcome and resource utilization deserves further studies
Thomas et al.^[52]^, 1992	Nasal CPAP	Oxygen by mask	14 / 14	59±4 / 55±10	14 / 14	IG: on the first day after surgery, 60 minutes of nasal CPAP with 5 cmH_2_O pressure CG: use of facial mask for oxygen therapy Outcomes: pain, pulmonary shunt, cardiac index	We conclude that the use of nasal CPAP is a simple, tolerable and effective method of treating hypoxemia in adult patients after coronary artery bypass surgery and warrants further study
Westerdahl et al.^[53]^, 2001	RVE, VEBH, EM, EULL, AM, blow bottle	RVE, VEBH, EM, EULL, AM, RI and PEP / RVE, VEBH, EM, EULL, AM	36 / 30 / 32	66±9.4 / 65.9±8.8 / 63.5±9.2	36 / 30 / 32	IG: EM, EULL, VEBH, AM. RVE in blow bottle , with 10 cm of water and plastic tube with 40 cm long and 1 cm in diameter, generating an expiratory resistance of 10 (± 1) cmH_2_O. 30 replicates were performed for RVE every hour during the day IG: EM, EULL, VEBH, AM. RVE through a face mask connected to a T tube with PEP 10 cmH_2_O and RI -5cmH_2_O. Thirty replicates were performed for RVE every hour during the day CG: EM, EULL, VEBH, AM and RVE without any device. Thirty replicates were performed for RVE every hour during the day Outcomes: VC, inspiratory capacity, FEV1, RV, TLC, diffusing capacity , pain, atelectasis, pleural effusion	No major differences among the treatment groups were found, but the impairment in pulmonary function tended to be less marked using the blow bottle technique. The blow bottle group had signicantly less reduction in total lung capacity compared to the deep breathing group, while the IR-PEP group did not signi. cantly differ from the other two groups.
Westerdahl et al.^[54]^, 2005	VEBH, EM, AM, PE	RVE, VEBH, EM, AM, PE, blow bottle	48 / 42	66±9 / 65±9	36 / 31	IG: care given once or twice daily for the first four days. EM, VEBH, PE and AM CG: PE, EM, VEBH, AM. Three sets of 10 repetitions of RVE were carried through every hour during the day, in the first four days. RVE in the blow bottle, with 10 cm of water and plastic tube with 50 cm long and 1 cm in diameter, generating an expiratory resistance 10 cmH_2_O Outcomes: VC, FVC, FEV1, inspiratory capacity, residual functional capacity, TLC, atelectasis, PaO_2_, PaCO_2_, pain, IMV time	Patients performing deep-breathing exercises after CABG surgery had significantly smaller atelectatic areas and better pulmonary function on the fourth postoperative day compared to a control group performing no exercises
Westerdahl et al.^[55]^, 2003	RVE	RVE, blow bottle/RVE, RI and PEP	21 / 20 / 20	66±9 / 64±8 / 64±10	18 / 16 / 15	IG: three sets of 10 repetitions of RVE without any device IG: three sets of 10 repetitions of RVE in the blow bottle, with 10 cm of water and plastic tube with 50 cm long and 1 cm in diameter, generating an R_E_ 10 cmH_2_O CG: three sets of 10 repetitions of RVE through a face mask connected to a T tube with PEP 15 cmH_2_O and R_I_ -5 cmH_2_OOutcomes: atelectasis, PaO_2_, PaCO_2_, IMV time	A significant decrease of atelectatic area,increase in aerated lung area and a small increase in PaO_2_ were found after performance of 30 deep breaths.No difference among the three breathing techniques was found

AR - alveolar recruitment; AM - ambulation; BPAP - bilevel positive pressure airway; CAR - active cycle of breathing; CG - control group; CPAP - continuous positive airway pressure; Cstat - static compliance; CV - current volume; EE - ends exercises; EM - early mobilization; EULL - exercise upper and lower limbs; FEV1 - forced expiratory volume in one second FiO_2_ - inspiratory oxygen fraction; FVC - forced vital capacity; ICU - intensive care unit; IG - intervention group; IMV - invasive mechanical ventilation; IMT - inspiratory muscle training; IS - incentive spirometry; LA -ladder; MH - manual hyperinflation; MV - minute volume; NBL - nebulization; NC - nasal catheter; NIV - non-invasive ventilation; NPI - none physiotherapy intervention; PaCO_2_- partial pressure of carbon dioxide; PaO_2_ - partial pressure of oxygen; PC-CMV - Pressure-control continuous mandatory ventilation; PD - postural drainage; PE - preoperative education; PEmax - maximal expiratory pressure; PEEP - positive end-expiratory pressure; PEF - peak expiratory flow; PEP - positive expiratory pressure; PImax - maximal inspiratory pressure; PRVC - pressure regulated volume control; PWC - progressive walking circuit; RI -inspiratory resistance; RCP - routine chest physiotherapy; RE - expiratory resistance; RPPI - intermittent positive pressure breathing; RV - residual volume; RVE - re-expansive ventilatory exercises; SA - support adapted; SIMV - synchronized intermittent mandatory ventilation; SMI - sustained maximal inspirations; 6MWD - six-minute walk distance; SP - support pressure; SpO_2_ - peripheral oxygen saturation; TLC - total lung capacity; TM - thoracic maneuvers; TS - tracheal suctioning; VC-CMV - volume-control continuous mandatory ventilation; VEBH - ventilatory exercises for bronchial hygiene; VC - vital capacity; VIPP - ventilation with intermittent positive pressure; VS - volume support; ZEEP - zero end expiratory pressure; WP - weaning protocol.

### CONSORT Statement

According to the CONSORT assessment, the three items that were best and worst described were, respectively: introduction (100%), interventions (100%), and outcomes and estimation (100%); allocation concealment (7.69%), ancillary analysis (7.69%), and generalizability (2.56%) (Table 3). The CONSORT extension (Table 4) presented as the best described items: participants (100%), interventions (100%), and components of the interventions (100%). On the other hand, the lowest scoring items were title and abstract (0%), assessment of adherence with the protocol (0%), and concealment method (5.12%).

**Table 3:** CONSORT *Statement*.

Author, Year	Title and introduction		Methods										Results							Discussion			Otherinformation		
	1	2	3	4	5	6	7	8	9	10	11	12	13	14	15	16	17	18	19	20	21	22	23	24	25
Al Jaaly et al.^[17]^, 2013	A	A	A	A	A	A	A	A	A	A	A	A	A	A	A	A	A	A	A	A	I	A	A	I	I
Barros et al.^[18]^, 2010	I	A	A	A	A	A	A	A	I	A	I	A	I	I	I	I	A	I	I	A	I	A	I	I	I
Blattner et al.^[19]^, 2008	A	A	A	A	A	A	A	A	A	A	A	A	A	A	A	A	A	I	I	A	I	A	I	I	I
Borges et al.^[20]^, 2013	I	A	A	A	A	A	I	A	I	I	I	A	I	A	A	A	A	I	I	I	I	A	I	I	I
Borghi-Silva et al.^[21]^, 2005	I	A	A	A	A	A	A	A	I	I	I	A	I	I	A	A	A	I	I	A	I	A	I	I	I
Castellana et al.^[22]^, 2003	I	A	I	A	A	A	I	I	I	I	I	A	I	I	A	I	A	A	I	I	I	A	I	I	I
Celebi et al.^[23]^, 2008	I	A	I	A	A	A	A	A	I	I	A	A	I	I	A	A	A	I	I	A	I	A	I	I	I
Crowe and Bradley^[24]^, 1997	I	A	I	A	A	A	I	A	I	I	A	A	I	A	A	A	A	A	I	I	I	A	I	I	I
Dongelmans et al.^[25]^, 2009	A	A	A	A	A	A	A	A	I	I	A	A	I	A	A	A	A	I	I	A	I	A	I	I	I
El-Kader^[26]^, 2011	I	A	I	A	A	A	I	I	I	I	I	A	I	I	A	I	A	I	A	I	I	A	I	I	I
Ferreira et al.^[27]^, 2010	I	A	A	A	A	A	I	I	I	I	I	A	I	I	A	A	A	I	I	A	A	A	I	I	A
Franco et al.^[28]^, 2011	I	A	I	A	A	A	I	I	I	I	I	I	I	I	I	A	A	I	I	I	I	A	I	I	I
Garcia and Costa^[29]^, 2002	I	A	I	A	A	A	I	A	I	I	I	I	I	A	I	I	A	I	I	I	I	A	I	I	I
Gust et al.^[30]^, 1996	I	A	I	I	A	A	I	I	I	I	I	A	I	I	A	A	A	I	I	I	I	A	I	I	I
Haefener et al.^[31]^, 2008	I	A	A	A	A	A	A	A	I	I	A	A	A	I	A	A	A	I	A	A	I	A	I	I	A
Hendrix et al.^[32]^, 2006	A	A	I	I	A	A	A	A	I	I	I	A	I	A	A	A	A	I	I	A	I	A	I	I	I
Herdy et al.^[33]^, 2008	A	A	I	A	A	A	A	I	I	I	A	A	I	A	A	A	A	I	I	I	I	A	I	I	I
Hirschhorn et al.^[34]^, 2008	A	A	A	A	A	A	A	A	A	A	A	A	A	A	A	A	A	I	A	A	I	A	I	I	I
Jenkins et al.^[35]^, 1989	I	A	A	I	A	A	I	I	I	I	A	A	I	I	A	A	A	I	I	I	I	A	I	I	A
Johnson et al.^[36]^, 1995	I	A	I	A	A	A	I	I	I	I	A	A	A	A	A	I	A	I	I	I	I	A	I	I	I
Marvel et al.^[37]^, 1986	I	A	I	I	A	A	I	A	I	I	A	A	I	A	A	I	A	I	I	I	I	A	I	I	I
Matheus et al.^[38]^, 2012	I	A	I	A	A	A	I	A	I	I	I	A	I	A	A	A	A	I	I	A	I	A	I	I	I
Matte et al.^[39]^, 2000	I	A	I	I	A	A	I	I	I	I	A	A	I	I	A	I	A	I	I	I	I	A	I	I	I
Mendes et al.^[40]^, 2010	A	A	A	I	A	A	A	A	I	I	I	A	A	I	A	A	A	I	A	A	I	A	I	I	I
Michalopoulos et al.^[41]^, 1998	I	A	I	A	A	A	A	I	I	I	I	A	I	I	A	I	A	I	I	I	I	A	I	I	I
Muller et al.^[42]^, 2006	I	A	A	A	A	A	I	I	I	I	I	A	I	A	A	A	A	I	I	I	I	A	I	I	I
Oikkonen et al.^[43]^, 1991	I	A	I	I	A	A	I	I	I	I	A	A	I	I	A	A	A	I	I	I	I	A	I	I	I
Renault et al.^[44]^, 2009	I	A	A	A	A	A	I	A	I	I	I	A	I	I	A	A	A	I	I	I	I	A	I	I	I
Richter Larsen et al.^[45]^, 1995	A	A	I	I	A	A	I	I	I	I	A	A	I	I	I	I	A	I	A	I	I	A	I	I	I
Romanini et al.^[46]^, 2007	I	A	I	A	A	A	I	A	I	I	I	A	I	A	I	I	A	I	I	A	I	A	I	I	A
Savci et al.^[47]^, 2011	A	A	I	I	A	A	A	A	I	I	I	A	A	I	A	A	A	I	I	A	I	A	I	I	I
Savci et al.^[48]^, 2006	I	A	I	A	A	A	I	I	I	I	A	A	I	A	A	A	A	I	I	I	I	A	I	I	I
Stein et al.^[49]^, 2009	I	A	A	A	A	A	A	A	I	I	A	A	A	I	A	A	A	I	A	A	I	A	A	I	A
Stiller et al.^[50]^, 1994	I	A	I	A	A	A	I	A	I	I	A	A	I	I	A	A	A	I	I	I	I	A	I	I	I
Sulzer et al.^[51]^, 2001	I	A	I	A	A	A	I	I	I	I	I	A	I	A	A	A	A	I	I	A	I	A	I	I	I
Thomas et al.^[52]^, 1992	I	A	I	I	A	A	I	I	I	I	I	A	I	I	A	I	A	I	I	I	I	A	I	I	I
Westerdahl et al.^[53]^, 2001	I	A	I	I	A	A	I	A	I	I	A	A	I	I	A	A	A	I	I	I	I	A	I	I	I
Westerdahl et al.^[54]^, 2005	I	A	I	A	A	A	A	I	I	I	A	A	I	I	A	A	A	I	I	I	I	A	I	I	A
Westerdahl et al.^[55]^, 2003	I	A	I	A	A	A	I	I	I	I	A	A	I	I	A	A	A	I	I	I	I	A	I	I	I

A - adequate; I - inadequate.

**Table 4:** Extension of CONSORT Statement.

Author, Year	Title and abstract	Methods										Results			Discussion	
	1	2	3	4	5	6	7	8	9	10	11	12	13	14	15	16
Al Jaaly et al.^[17]^, 2013	I	A	A	A	A	I	A	A	I	I	A	A	A	A	I	I
Barros et al.^[18]^, 2010	I	A	A	A	I	I	A	A	I	I	A	I	A	I	I	I
Blattner et al.^[19]^, 2008	I	A	A	A	A	I	A	A	A	A	A	A	A	A	I	I
Borges et al.^[20]^, 2013	I	A	A	A	I	I	I	A	I	I	A	I	A	A	I	I
Borghi-Silva et al.^[21]^, 2005	I	A	A	A	A	I	I	A	I	I	A	I	A	A	I	I
Castellana et al.^[22]^, 2003	I	A	A	A	I	I	I	I	I	I	A	I	A	A	I	I
Celebi et al.^[23]^, 2008	I	A	A	A	I	I	A	A	A	I	A	I	A	A	I	I
Crowe and Bradley^[24]^, 1997	I	A	A	A	A	I	I	A	A	I	A	I	A	A	I	I
Dongelmans et al.^[25]^, 2009	I	A	A	A	A	I	A	A	A	I	A	I	A	A	I	I
El-Kader^[26]^, 2011	I	A	A	A	I	I	I	I	I	I	A	I	A	A	I	I
Ferreira et al.^[27]^, 2010	I	A	A	A	I	I	I	I	I	I	A	I	A	A	I	I
Franco et al.^[28]^, 2011	I	A	A	A	I	I	I	I	I	I	I	I	A	I	I	I
Garcia and Costa^[29]^, 2002	I	A	A	A	I	I	I	A	I	I	A	I	A	I	I	I
Gust et al.^[30]^, 1996	I	A	A	A	A	I	I	I	I	I	A	I	A	A	I	I
Haefener et al.^[31]^, 2008	I	A	A	A	A	I	A	A	A	I	A	A	A	A	I	I
Hendrix et al.^[32]^, 2006	I	A	A	A	A	I	A	A	I	I	A	I	A	A	I	I
Herdy et al.^[33]^, 2008	I	A	A	A	A	I	A	I	A	I	A	I	A	A	I	I
Hirschhorn et al.^[34]^, 2008	I	A	A	A	A	I	A	A	A	A	A	A	A	A	I	I
Jenkins et al.^[35]^, 1989	I	A	A	A	I	I	I	I	A	I	A	I	A	A	I	I
Johnson et al.^[36]^, 1995	I	A	A	A	I	I	I	I	A	I	A	A	A	A	I	I
Marvel et al.^[37]^, 1986	I	A	A	A	A	I	I	A	A	I	A	I	A	A	I	I
Matheus et al.^[38]^, 2012	I	A	A	A	I	I	I	A	I	I	A	I	A	A	I	I
Matte et al.^[39]^, 2000	I	A	A	A	I	I	I	I	A	I	A	I	A	A	I	I
Mendes et al.^[40]^, 2010	I	A	A	A	I	I	A	A	I	I	A	A	A	A	I	I
Michalopoulos et al.^[41]^, 1998	I	A	A	A	A	I	A	I	I	I	A	I	A	A	I	I
Muller et al.^[42]^, 2006	I	A	A	A	I	I	I	I	I	I	A	I	A	A	I	I
Oikkonen et al.^[43]^, 1991	I	A	A	A	I	I	I	I	A	I	A	I	A	A	I	I
Renault et al.^[44]^, 2009	I	A	A	A	I	I	I	A	I	I	A	I	A	A	I	I
Richter Larsen et al.^[45]^, 1995	I	A	A	A	I	I	I	I	A	I	A	I	A	I	I	I
Romanini et al.^[46]^, 2007	I	A	A	A	I	I	I	A	I	I	A	I	A	I	I	I
Savci et al.^[47]^, 2011	I	A	A	A	A	I	A	A	I	I	A	A	A	A	I	I
Savci et al.^[48]^, 2006	I	A	A	A	A	I	I	I	A	I	A	I	A	A	I	I
Stein et al.^[49]^, 2009	I	A	A	A	I	I	A	A	A	I	A	A	A	A	I	I
Stiller et al.^[50]^, 1994	I	A	A	A	I	I	I	A	A	I	A	I	A	A	I	I
Sulzer et al.^[51]^, 2001	I	A	A	A	A	I	I	I	I	I	A	I	A	A	I	I
Thomas et al.^[52]^, 1992	I	A	A	A	I	I	I	I	I	I	A	I	A	A	I	I
Westerdahl et al.^[53]^, 2001	I	A	A	A	I	I	I	A	A	I	A	I	A	A	I	I
Westerdahl et al.^[54]^, 2005	I	A	A	A	I	I	A	I	A	I	A	I	A	A	I	I
Westerdahl et al.^[55]^, 2003	I	A	A	A	A	I	I	I	A	I	A	I	A	A	I	I

A - adequate; I - inadequate.

**Fig. 2:**
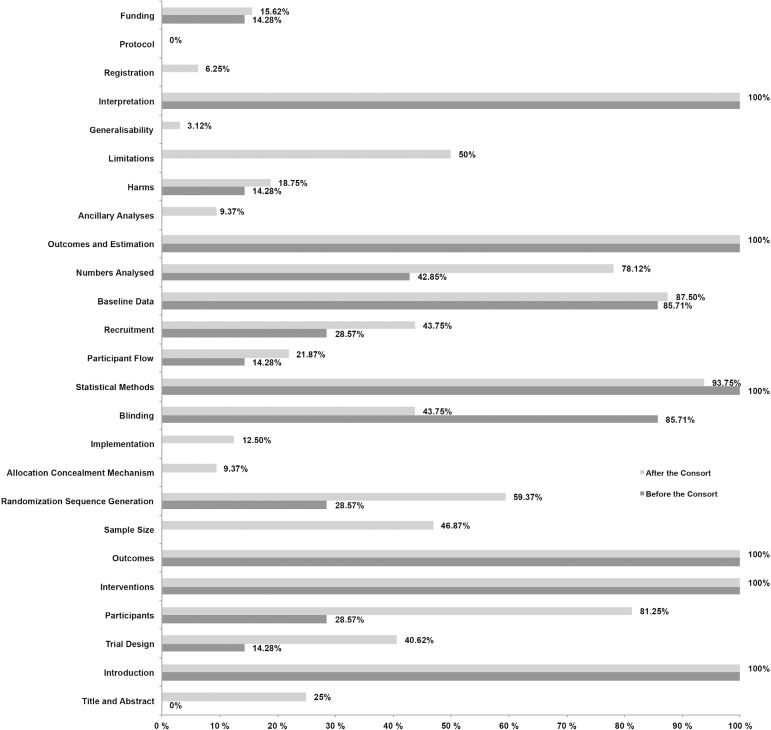
Comparison studies published before and after the CONSORT Statement.

Seven studies conducted before the CONSORT publication were identified. When compared to other studies, the items introduction, interventions, results, outcomes and estimation, interpretation, and protocol remained equally adequate. The correct description of the items blinding and statistical methods decreased 41.96% and 6.25% respectively in the studies published after the CONSORT. All of the 17 remaining items were described more frequently after the CONSORT publication, as follows: title and abstract (increase of 10.72%), design (increase of 26.34%), participants (increase of 52.68%), sample size (no description of this item was found in any of the studies published previously to the CONSORT, but it was described in 46.87% of the studies after it), random sequence generation (increase of 30.80%), allocation concealment (no description of this item was found in any of the studies published previously to the CONSORT, but it was described in 9.37% of the studies after it), allocation implementation (no description of this item was found in any of the studies published previously to the CONSORT, but it was described in 12.50% of the studies after it), participant flow diagram (increase of 6.69%), recruitment (increase of 15.18%), characteristics (increase of 1.79%), numbers analyzed (increase of 35.27%), ancillary analyses (no description of this item was found in any of the studies published previously to the CONSORT, but it was described in 9.37% of the studies after it), harms (increase of 4.47%), limitations (no description of this item was found in any of the studies published previously to the CONSORT, but it was described in 50% of the studies after it), generalizability (no description of this item was found in any of the studies published previously to the CONSORT, but it was described in 3.12% of the studies after it), registration (no description of this item was found in any of the studies published previously to the CONSORT, but it was described in 6.25% of the studies after it) and funding (increase of 1.34%). The item "protocol" was not appropriate according to the CONSORT requirements in any of the studies evaluated (Figure 2).

**Fig. 3:**
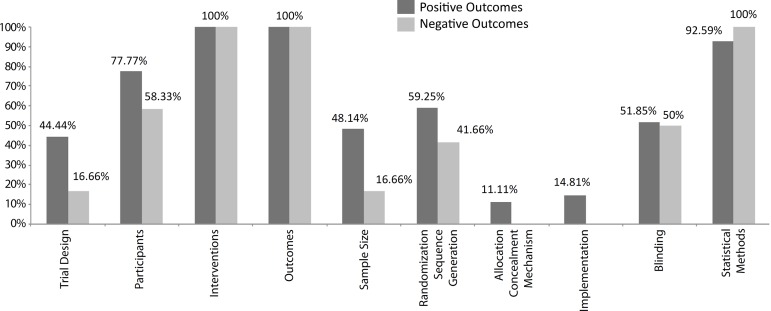
Methods section of CONSORT: comparison studies with positive and negative outcomes.

Among the 39 studies, 27 presented its final outcomes as positive and 12 as negative with the proposed treatment. Regarding the CONSORT checklist's Methods section, when evaluated separately in accordance with the outcome, all items showed to have equal or better methodological quality in the studies with positive outcomes, except for the Statistical Methods item (Figure 3).

### Risk of Bias

Regarding the assessment of the Cochrane Collaboration's tool for risk of bias, description of losses and exclusions in 66.66% of the studies, proper random sequence generation in 51.28%, blinding of outcome assessors in 46.15%, intention-to-treat analysis in 12.82%, and allocation concealment and blinding of patients and investigators in 7.69% could be noted (Table 5).

**Table 5:** Risk of bias.

Author, Year	Adequate sequence generation	Allocation concealment	Blinding of patients and investigators	Blinding of outcome assessors	Description of losses and exclusions	Intention-to-treat analysis
Al Jaaly et al.^[17]^, 2013	Yes	Yes	No	Yes	Yes	Yes
Barros et al.^[18]^, 2010	Yes	No	No	No	Yes	No
Blattner et al.^[19]^, 2008	Yes	Yes	No	Yes	Yes	Yes
Borges et al.^[20]^, 2013	Yes	No	No	No	Yes	No
Borghi-Silva et al.^[21]^, 2005	Yes	No	No	No	Yes	No
Castellana et al.^[22]^, 2003	No	No	No	No	No	Not report
Celebi et al.^[23]^, 2008	Yes	No	No	Yes	No	Not report
Crowe and Bradley^[24]^, 1997	Yes	No	No	Yes	No	Not report
Dongelmans et al.^[25]^, 2009	Yes	No	Yes	No	Yes	No
El-Kader^[26]^, 2011	No	No	No	No	No	Not report
Ferreira et al.^[27]^, 2010	No	No	No	No	No	Not report
Franco et al.^[28]^, 2011	No	No	No	No	No	Not report
Garcia and Costa^[29]^, 2002	No	No	No	No	Yes	No
Gust et al.^[30]^, 1996	No	No	No	No	No	Not report
Haefener et al.^[31]^, 2008	Yes	No	No	Yes	Yes	Yes
Hendrix et al.^[32]^, 2006	Yes	No	No	No	No	Not report
Herdy et al.^[33]^, 2008	No	No	No	Yes	Yes	Yes
Hirschhorn et al.^[34]^, 2008	Yes	Yes	Yes	Yes	Yes	Yes
Jenkins et al.^[35]^, 1989	No	No	Yes	No	Yes	No
Johnson et al.^[36]^, 1995	No	No	No	Yes	Yes	No
Marvel et al.^[37]^, 1986	Yes	No	No	Yes	Yes	No
Matheus et al.^[38]^, 2012	Yes	No	No	No	No	Not report
Matte et al.^[39]^, 2000	No	No	No	Yes	Yes	No
Mendes et al.^[40]^, 2010	Yes	No	No	No	Yes	No
Michalopoulos et al.^[41]^, 1998	No	No	No	No	Yes	No
Muller et al.^[42]^, 2006	No	No	No	No	No	Not report
Oikkonen et al.^[43]^, 1991	No	No	No	Yes	Yes	No
Renault et al.^[44]^, 2009	Yes	No	No	No	Yes	No
Richter Larsen et al.^[45]^, 1995	No	No	No	Yes	Yes	No
Romanini et al.^[46]^, 2007	Yes	No	No	No	No	Not report
Savci et al.^[47]^, 2011	Yes	No	No	No	Yes	No
Savci et al.^[48]^, 2006	No	No	No	Yes	No	Not report
Stein et al.^[49]^, 2009	Yes	No	No	Yes	Yes	No
Stiller et al.^[50]^, 1994	Yes	No	No	Yes	Yes	No
Sulzer et al.^[51]^, 2001	No	No	No	No	Yes	No
Thomas et al.^[52]^, 1992	No	No	No	No	No	Not report
Westerdahl et al.^[53]^, 2001	Yes	No	No	Yes	Yes	No
Westerdahl et al.^[54]^, 2005	No	No	No	Yes	Yes	No
Westerdahl et al.^[55]^, 2003	No	No	No	Yes	Yes	No

## DISCUSSION

The development of research related to the assessment of the methodological quality of scientific production in health, especially in physiotherapy, is still of little expression. Therefore, this is the first systematic review that has assessed the methodological quality of RCT of physiotherapy treatment in postoperative patients of CABG in the ICU based on the instruments CONSORT Statement, its extension for non-pharmacologic treatment interventions and the Cochrane Collaboration's tool for assessing risk of bias.

In general, over the years, the methodological quality of studies has increased, especially if we set as a cutoff the year of publication of the CONSORT Statement checklist. Among the checklist's 25 items, five have remained with an equal adequacy rate and 17 have been more broadly documented. Geha et al.^[10]^ when assessing the quality of cardiorespiratory physiotherapy studies, found similar results, with a rising curve of quality assessed through the PEDro scale. In a study published by Hopewell et al.^[56]^, in which the quality of trials indexed by the PubMed published between 2000 and 2006 were evaluated, the results were very similar. While the quality of the studies had improved over time, it was still below an acceptable level (for example, only 45% of the trials had included a calculation of the sample size). This suggests that, despite the release of the CONSORT Statement over the last decade, a large proportion of authors, reviewers and journal editors have not yet implemented these recommendations.

The two items that showed an adequacy decline were statistical methods and blinding. The first demonstrated a difference smaller than 7% (two studies), being therefore irrelevant. In studies published after the CONSORT, a reduction of the reporting of blinding in 41.96% of the studies was observed, and only 43.75% informed that blinding was performed in their methodology, with no further details. When the evaluation was directed at whom was blinded (patient, investigators or outcome assessor), the adequacy was even lower, reaching 7.69%. Our results are similar to the studies^[8-11]^ who assessed the quality of studies in the areas of cardiothoracic, neurological, sports and aquatic physiotherapy, respectively. Research indicates that blinding, or lack thereof, is associated with a greater tendency to maximize the treatment's effect^[57-61]^. In a study by Boutron et al.^[62]^, in which pharmacologic and non-pharmacologic treatments for hip or knee osteoarthritis were compared, blinding was found to be less frequent in nonpharmacologic studies, even when there is a possibility to do it. It should be emphasized that an adequate methodological conduct in relation to blinding results in a higher number of professionals involved and often adds costs to the research, which becomes a limiting factor. The lack of blinding interferes directly on the results, making both its internal and external validity look dubious. Consequently, the use of these studies in systematic reviews becomes limited, generating biased results.

Due to the large number of publications, the standardization of papers to the rules of each journal must be followed, which mainly includes a limit for the number of words, tables and figures. For this reason, very precise details of the research development may end up without space. Given this reality, none of the papers included in this review presented the items title and abstract, assessment of adherence to the protocol, interpretation, and generalizability as required by the CONSORT extension for non-pharmacologic treatment interventions. However, these undescribed data may have been part of the research development, but they were not disclosed. Specifically, there is no available information in the literature for us to corroborate such finding. A combination of techniques was present in 69.23% of the studies. This result is in accordance with a systematic review published by Stiller^[63]^ on physiotherapy performance in the ICU. It was not possible to evaluate the effectiveness of each technique alone, the same way as the large heterogeneity of methodologies and samples made it impossible to carry out a statistical analysis.

Another interesting finding of our research was that the 27 studies with positive outcomes demonstrated a better quality regarding the 10 items of CONSORT Methods section. Except for the statistical methods, in which the difference was of only 7%, all other items were appropriately described more often in studies with positive outcomes. Beckerman et al.^[64]^, when evaluating laser therapy in different musculoskeletal and dermatological conditions, found similar results, with studies with positive outcomes having better quality. A year later, the same author found contrary results when assessing the effectiveness of physiotherapy in musculoskeletal disorders^[65]^. Studies with negative outcomes tend to be submitted less frequently, with a lower acceptance by journal reviewers. Therefore, there may be an overestimation of treatment effects, leading to important implications in choosing the best treatment to follow.

The gap between the publication of the results of a scientific research and its actual implementation in the professional routine is still substantial, leading to health care practices of levels lower than expected^[66]^. However, prior to this, the research planning and development should be improved so that its results are as close as possible to the truth and are legitimized by a methodology of quality.

### Limitation of the Study

A limitation of this systematic review is that literature search was not conducted in Embase database.

## CONCLUSION

The description of the necessary items for the correct execution, conduction and publication of studies has increased over the years, but it still has great scope for improvement. In general, the methodological quality is below an acceptable level in order to obtain results that are reliable and applicable in the daily practice.

**Table:** 

Authors' roles & responsibilities	
JL	Conception and design of the work; acquisition, analysis, interpretation of data for the work; drafting the work and revising it critically for important intellectual content; final approval of the version to be published
CS	Conception and design of the work; acquisition, analysis, interpretation of data for the work; drafting the work and revising it critically for important intellectual content; final approval of the version to be published
RDMP	Conception and design of the work; acquisition, analysis, interpretation of data for the work; drafting the work and revising it critically for important intellectual content; final approval of the version to be published
